# Evaluation of the frequency and predictive factors of psychotropic drugs consumption among students of the university of pharmacy of monastir

**DOI:** 10.1192/j.eurpsy.2021.1523

**Published:** 2021-08-13

**Authors:** N. Bensaid, A. Ghorbel, M. Rekik, N. Messedi, S. Smaoui, S. Hachicha, F. Messadi, J. Aloulou

**Affiliations:** 1 Class Toxicology Unit, hygiene Loboratory, sfax, Tunisia; 2 Toxicology Unit, hygiene Loboratory, sfax, Tunisia; 3 Psychiatry (b), Hedi Chaker University hospital, sfax, Tunisia; 4 Microbiology, hygiene laboratory, sfax, Tunisia; 5 Microbiology Unit, hygiene Loboratory, sfax, Tunisia

## Abstract

**Introduction:**

Psychotropic medications are widely used in Tunisia. Studies about frequency of substance use are rare

**Objectives:**

The purpose of this work is to determine frequency of use of psychotropic medication among pharmacy students in Monastir University during their university years and during the last year and to assess the factors associated to this consumption

**Methods:**

A retrospective study was used to collect the information about 145 participants using a questionnaire asking about the consumption of psychotropic medications and the factors associated to substance use

**Results:**

145 subjects aged 22.71 years +/- 2.04 among them 25% was men and 75 % women. Psychotropic medication use was 20 % during university years and 17.24 % during the last year. 45 % of the users had their medications without a prescription from a doctor. 17 % of the users of psychotropic medication weren’t informed about the effects of the drugs. A higher consumption of psychotropic drugs was observed among older individuals (p=0.009; F=6.928), redoubling individuals (p=0.003), with conflictive relationships with family (p=0.001), using others psychotropic substances, feeling often nervous (p=0.003; F=9.318) and with personal history of mental illness (p=0.002).

**Conclusions:**

Our finding underlines the need of larger more comprehensive surveys to determine the efficiency of the prevention strategies and to minimize the consumption of psychotropic drugs or to control it.

**Keyword:**

dependence Risk factors drugs students

